# Sol-Gel Synthesis, *in vitro* Behavior, and Human Bone Marrow-Derived Mesenchymal Stem Cell Differentiation and Proliferation of Bioactive Glass 58S

**DOI:** 10.52547/ibj.25.3.180

**Published:** 2021-02-28

**Authors:** Majid Rastegar Ramsheh, Aliasghar Behnamghader, Ali Khanlarkhani

**Affiliations:** Department of Nanotechnology and Advanced Materials, Materials and Energy Research Center, Karaj, Iran

**Keywords:** Bioactive glass 58S, Gene expression, Mesenchymal stem cells

## Abstract

**Background:**

Bioactive glasses 58S, are silicate-based materials containing calcium and phosphate, which dissolved in body fluid and bond to the bone tissue. This type of bioactive glass is highly biocompatible and has a wide range of clinical applications.

**Methods:**

The 58S glass powders were synthesized *via* sol-gel methods, using tetraethyl orthosilicate, triethyl phosphate, and calcium nitrate, as precursors. Upon the analyses of phase and chemical structures of bioactive glass in different gelation times (12, 48, and 100 h), the appropriate heat treatment (at 525, 575, and 625 °C) was performed to eliminate nitrate compounds and stabilize the glass powder samples. The *in vitro* assay in SBF solution revealed the bioactivity of the synthesized 58S glass through the morphological (SEM), chemical structure (FTIR), release of calcium, phosphorous and silicon elements, pH variations, and weight loss measurements. The behavior of MSCs in the presence of bioactive glass powders was studied by MTT cytotoxicity, cell staining, ALP activity and biomineralization tests, as well as by the evaluation of *ALP*, *osteocalcin*, *osteonectin*, *collagen* *I*, and *RUNX2* gene expression.

**Results:**

The results confirmed a gelation time of 100 h and a calcination temperature of 575 °C at optimal conditions for the synthesis of nitrate-free bioactive glass powders.

**Conclusion:**

The glass spherical nanoparticles in the range of 20-30 nm possess the improved bioactivity and osteogenic properties as demanded for bone tissue engineering.

## INTRODUCTION

A considerable part of the health problems arises from bone defects, resulting in the development of orthopedic market with a value of $4.3 billion in 2015, which will expectedly approach $46.5 billion by 2024^[^^1^^-^^3^^]^. To resolve such difficulties, orthopedic surgeons and investigators have introduced various approaches, each with some benefits and drawbacks^[^^4^^]^. Bone graft is a reliable therapeutic technique utilized in their entire forms, namely autographs, allografts, xenografts, and synthetic grafts, for repairing and replacing bone defects. Autografts, identified as a golden standard for repairing bone defects, have exhibited outstanding biological features, but these grafts have limitations owing to the morbid state of the donor and inadequate supply. Allografts/xenografts are sufficiently accessible but have restricted applications due to the threat of contaminations and loss of osteogenic ability. Recently, synthetic grafts fabricated by bioactive materials have received considerable interest for their applications in bone tissue repair and regeneration. This matter is attributable to the potential properties of the grafts, including superb bioactivity, osteo-conductivity, resorbability, and the capability of fostering the growth of bone cells^[^^5^^-^^10^^]^.

Following the initial introduction by Hench^[^^11^^]^, bioactive glasses were appealing substances for bone graft uses because of their multipurpose features, such as the improved revascularization capacity, osteoblast adhesion, enzyme activity, and differentiation of MSCs and osteoprogenitor cells^[^^12^^-^^15^^]^. Additionally, bioactive glasses are able to create a robust chemical bond with hard and soft tissues with no intervening fibrous layer, which results in an intimate link between bone and glass. According to reports from *in vivo* investigations, bioactive glass compositions generate no local or systemic toxicity, no inflammation, and no foreign body response during implantation. Such features render bioactive glass-based materials a fascinating candidate for biomedical application purposes^[^^12^^,^^13^^,^^16^^]^.

After the early detection of bioactive glass composition, identified as 45S5 Bioglass^®^ (45% SiO_2_, 24.5% CaO, 24.5% Na_2_O, and 6% P_2_O_5_ [wt.%]), a large body of related investigations have led to the development of diverse bioactive glass formulations^[^^12^^,^^13^^]^. Based on ternary system SiO_2_–CaO–P_2_O_5_, Glasses comprise a major group of materials with extensive biomedical applications, which display fascinating biological attributes, including bioactivity, biodegradability, and bone-bonding capacity, rendering them potential options for hard tissue engineering^[^^17^^-^^19^^]^.

For the production/synthesis of bioactive glasses, melting and sol–gel techniques are two common procedures with wide applications. Melting is a simple and suitable method for traditional massive glass production at elevated temperatures (>1350 °C). In comparison to melting, the sol–gel method is a chemical procedure that is advantageous in terms of a rather low reaction temperature, which enables to synthesize glasses with greater purity, homogeneity, and nanoscale structure^[^^12^^,^^20^^]^. Moreover, simplified glass compositions, including ternary glass composition (SiO_2_–CaO–P_2_O_5_), are obtainable through sol–gel path as it excludes adding sodium oxide used for lowering melting temperature^[^^20^^,^^21^^]^.

The present research investigated the synthesis of bioactive glass nanoparticles with the composition of SiO_2_−CaO−P_2_O_5_ through sol–gel technique. So far, many publications have been released to discuss the bioactive glass preparation n using sol-gel method. Nanosized 58S bioactive glass particles were synthesized by a three-dimensional ordered macroporous carbon template with a pore size of 400 nm^[^^21^^]^. The obtained 58S bioactive glass particles possessed a diameter of 300 nm with narrow size distribution and uniform spherical morphology. The 58S particles are able to induce carbonated hydroxyapatite formation, revealing their outstanding bioactivity. Bui *et al.*^[^^22^^]^ prepared amorphous 58S bioglass with specific surface area (99.1 m^2^/g) by sol–gel process. The ammonia was used to facilitate the condensation reactions within an acidic solution prepared by tetraethyl orthosilicate, triethyl phosphate, and calcium nitrate tetrahydrate. *In vitro* experiments have confirmed the formation of a dense and visible bioactive hydroxyapatite layer on the surface of glass particles after two days, showing the improved bioactivity of the synthesized glass. Luz *et al.*^[^^23^^]^ synthesized SiO_2_–CaO–P_2_O_5_ bioactive glass nanoparticles via an optimized sol–gel method and evaluated the pH of preparation and the effect of heat treatment temperature. Round shaped particles with sizes below 50 nm were produced at pH 11.5. From the obtained data, it was concluded that the thermal treatment at 700 °C improved the bioactivity of the glass particles, being more effective when the nanoparticles were prepared at pH 11.5^[^^23^^]^. 

To the best of our knowledge, there is no report, at least a few published works, on the combined effect of gelation-calcination on the microstructure and *in vitro* behavior of 58S bioactive glass particles. Herein, for the first time, the effect of gelation and calcination temperature on the microstructure and chemical composition of the glasses were assessed by XRD, FESEM, EDS, and FTIR.* In vitro* examinations in SBF were performed on the bioactive glasses. Additionally, the biological features of the generated bioactive glass powders were studied by MTT assay, cell staining, ALP activity, and biomineralization tests. As another innovative aspect of the present research, we investigated the expression of bone-specific gene expression, i.e. *ALP*, *osteocalcin*, *osteonectin*, *collagen I*, and *RUNX2* by hMSCs in the vicinity of the synthesized 58S bioactive glass particles, which was not considered in previous studies^[^^24^^-^^26^^]^.

## MATERIALS AND METHODS

**Materials**

The starting chemicals, including TEOS (Si(OC_2_H_5_)_4_), TEO ((C_2_H_5_O)_3_P), calcium nitrate tetrahydrate (Ca(NO_3_)_2_.4H_2_O), ethyl alcohol (C_2_H_5_OH), and nitric acid (HNO_3_) materials were procured from Merck, Germany.

**Synthesis** **of bioactive glass nanoparticles**

Synthesis of SiO_2_–CaO–P_2_O_5_ (58-38-4% mol) bioactive glasses was carried out using TEOS, TEP, and calcium nitrate tetrahydrate precursors as silicon, phosphorus, and calcium resources, respectively. This process was performed at different gelation times of 12, 48, and 100 h and calcination temperature of 525, 575, and 625 °C. In a typical synthesis process, 200 ml of ethanol-deionized water (50:50) was first acidified to pH 2 using nitric acid (0.1 M). The solution was stirred in a 30 °C water bath at 300 rpm for 10 minutes, after which TEOS was added to the solution. TEP precursor and calcium nitrate powder were sequentially added following admixing for 35 and 40 minutes, respectively. The solution was then blended to obtain a transparent solution, followed by incubation at certain time/temperature to be converted into the gel. The prepared wet gel was dried at 150 °C for 48 h, then calcined at 525 °C temperatures for 2 h and finally pulverized by a planetary ball mill at 300 rpm for 3 h.

**Characterization of bioactive glass nanoparticles**

To achieve the optimal stabilization temperature, we analyzed the dried gels by concurrent thermogravimetry and differential thermal analysis (STA Instruments, BÄHR 503, Germany) at ambient temperature (1000 °C) under atmospheric condition with heating rates of 5 and 20 °C/min. Siemens‒D500 X-ray diffractometer equipped with a CuKα (λ = 1.542 Å) rotating anode, working at 40 kV and 30 Ma, was employed for the phase analysis of bioactive glass powders. The XRD patterns were collected over an angular range of 20°-70°, with a step-size of 0.02° and a scan speed of 2°/min. The functional group of the manufactured bioactive glass nanoparticles was examined on a Bruker-Vector 33 Fourier transfer infrared spectrophotometer over a wavenumber range of 400–4000 cm^-1^. The microstructure and morphology of the bioactive glass nanoparticles were analyzed by FESEM using the Tescan Mira 3 LMU electron microscope with an accelerating voltage of 15 kV. The elemental of the bioactive glass nanoparticles was investigated with EDS acquired on a Quantax 200 instrument, Germany.

***In vitro*** **bioactivity study**

*In vitro *bioactivity was assessed by 10 mg of glass powders in 10 ml of SBF in sterile polyethylene containers at 37 °C for seven days. The solution was refreshed every two days. After soaking for seven days, the samples were filtered, rinsed repeatedly by deionized water, and finally dried at room temperature. The surface of the samples was examined by means of SEM-EDS (AIS2300C, Seron Technologies, Korea) and FTIR (Spectrum 400, PerkinElmer, USA).

**MTT cytotoxicity assay**

hMSCs obtained from Nanobiotechnology Research Center, Baqiyatallah University of Medical Sciences (Tehran, Iran) were used to evaluate the biological behavior of the synthesized glass powders. The toxic property of the produced bioactive glass nanoparticles was evaluated by colorimetric MTT tetrazolium reduction assay. The cells were grown in 75-ml flasks by 12 ml of culture medium with high glucose concentration (DMEM-h, Gibco, Germany), together with 5% of fetal bovine serum (Gibco) and 1 wt%. of antibiotic (penicillin-streptomycin, Gibco). Afterwards, the flasks were incubated at 37 °C, 90% relative humidity, and 5% CO_2_. The culture medium was refreshed every two days to achieve a cell density of 90%. The cells were then passaged, detached from the bottom of the flask by 0.2% of Trypsin-EDTA (Gibco) and counted using a Neubauer Chamber. The hMSCs were seeded in a 96-well microplate (cell density of 1 × 10^4^ cells per well). The glass powders were disinfected by rinsing in 70 wt.% ethanol, irradiation by UV exposure for 20 min and autoclavation at 120 °C for 30 min. Thereafter, the samples were placed in the cell culture at the concentrations of 1.0, 2.0, 5.0, and 10.0 mg.mL^-1^. In a range of three time points, the supernatant was harvested, and cells were rinsed with PBS. Subsequently, 200 μl of the culture medium holding 20 μl of MTT solution (Sigma Aldrich) was added to each well, followed by incubation of the samples at 37 °C, 98% relative humidity, and 5% CO_2 _for 4 h. The supernatant was discarded, and the Formosan crystals were dissolved by the addition of 100 μl of dimethyl sulfoxide (Sigma Aldrich, USA). In the end, the optical absorbance of the resultant blue-violet solution (directly related to the number of metabolically active cells) was determined by an ELISA microplate reader (Sunrise, Tecan, Austria) at λ = 570 nm. Comparisons were made using a control sample (in the absence of FA nanoparticles). 

**Cell viability**

The cell viability was assessed by acridine orange staining test on the glass powders. To this end, the dual fluorescent staining solution (1 μl) comprising of acridine orange (100 μg/ml; Sigma Aldrich) was added to the cell culture well, followed by washing with PBS. Next, the well was observed using a fluorescent microscope (Leica 090-135002, Germany). 

**ALP activity**

ALP activity assay was performed at the intervals of 7 and 14 days. At the completion of each interval, 200 μl of Radioimmunoprecipitation assay buffer was added to the cell culture wells. The culture plates were then vortexed four times, each for 20 minutes. Then, the released Proteins were separated from the cells and nanomaterials were by centrifuging (21,382 ×g) at 4°C for 15 minutes. 50 μl of the supernatant was transferred to a tube, and then 150 μl of ALP solution with a solution ratio of (R1/R2) 4: 1 was added to the samples based on Paadko kit (Tehran, Iran). Lastly, ALP activity was determined using an ELISA microplate reader (Sunrise) at the wavelength of 450 nm.

**Alizarin red staining**

Alizarin Red staining test was carried out to examine the ability of nanomaterials in promoting the bone differentiation during 14 days. Afterwards, the cells were washed with PBS and fixed by 4% solution of formaldehyde (Merck, Germany) for 20 minutes. Next, the cells were rewashed with PBS and stained with 1% solution of Alizarin Red (Merck, Germany) for 5 minutes. Extra dye was removed by repeated washing using distilled water. Finally, the stained bone cells were visualized by a light microscope. As a negative control, the MSCs without a differentiation medium was stained in parallel.

**Calcium deposition**

The calcium deposition test was carried out by a procedure the same as ALP test, except that each sample was first washed with PBS, and then 200 μl of 6% HCL was added. Subsequently, the samples were relocated into vials, vortexed and stirred at ambient temperature for 40 minutes. The quantities of calcium precipitates were then determined by a calcium content kit (Sigma Aldrich). Finally, the optical absorbance was read by an ELISA reader apparatus at the wavelength of 570 nm.

**Gene expression**

Following 7 and 14 days, the differentiated cells were tested for bone-specific gene expression (*ALP*, *osteocalcin*, *osteonectin*, *collagen* *I*, and *RUNX2*). Cellular RNA of differentiated MSCs was extracted using a commercial RNA extraction kit (Thermo Scientific, USA). To remove the DNA, the extracted RNA samples were treated by DNase 1. Then the purity and concentration of the extracted RNA were estimated with a NanoDrop spectrophotometer. Thereafter, 5 μl of RNA was entered to cDNA synthesis reaction using a commercial cDNA synthesis kit and random hexamer primers (Thermo Scientific). For *reverse transcription polymerase chain reaction*, 1 μl of cDNA, 10 μl of low Rox Master mix real time (Ampliqon Denmark), and 1 μl of forward and reverse primers (*ALP*, *osteocalcin*, *osteonectin*, *collagen I*, and *RUNX2*) with a concentration of 10 pmol/μl were added to each reaction tube, along with adding double distilled water to a final volume of 20 μl. It is noteworthy that the expression of target genes was normalized against hypoxanthine-guanine phosophoribosyltransferase. The *reverse transcription polymerase chain reaction* thermal cycles (ABI 7500, USA) were as follows: an initial denaturation at 95 °C for 15 min, followed by 40 cycles of 95 °C for 20 seconds, 60 °C (annealing) for 30 seconds, and 72 °C (extension) for 30 seconds.

**Statistical analysis**

The experiments were conducted in triplicate, and the results were expressed as means ± SD. Mean values were regarded to be significantly different at *p* < 0.05.

## RESULTS AND DISCUSSION

**Characterization of the glass powder**

**Fig. 1:**
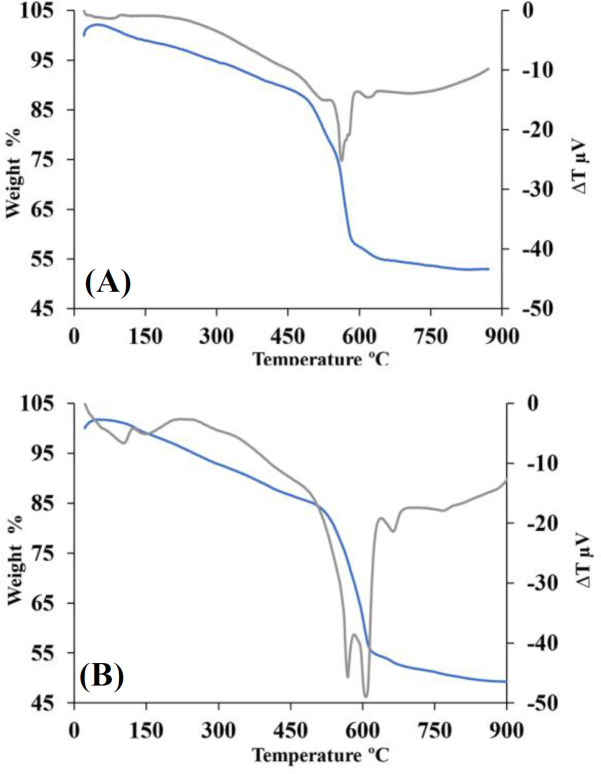
The STA curve of the dried gel at the heating rates of (A) 5 °C/min and (B) 20 °C/min

To study the thermal behavior of dried gel at greater heating rates, the samples were analyzed by STA at heating rates of 5 and 20 °C/min. As depicted in Figure 1, the thermal behaviors of the dried gel are completely the same at the two heating rates. The Figure displays a total weight drop of 45-50% after heating. Eliminating physically adsorbed water and alcohol molecules and triggering nitrate degradation reaction were observed up to 490 °C. In the temperature ranges 490 to 570 °C, free or attached nitrate compounds were released, and silanol groups were densificated. Two distinctive peaks at ~570 and 605 °C belonged to the termination of nitrate degradation and development of the first stable crystalline phases, respectively. The evaporation rates of the evaporative phases prior to the elimination of the nitrate compounds are almost 20-25%, followed by nearly 30-35%. An exothermic reaction peak in the *differential thermal analysis* curve situated at 620-650 °C characterizes the crystallization reaction of the glass^[^^22^^,^^23^^]^. The STA curves led to the conclusion that most of volatile substances eliminated to 600 °C. The weight loss occurred over ~600 °C was insignificant, confirming the elimination of organic remnants^[^^22^^,^^24^^]^.

**Fig. 2:**
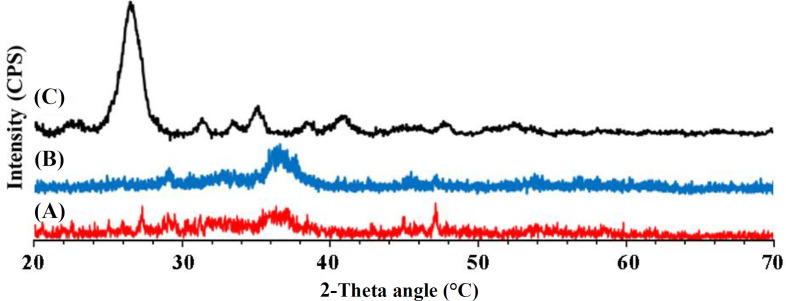
XRD patterns of the glass powder after heat treatment at (A) 525, (B) 575, and (C) 625 °C

The diffraction pattern of the glass powder following thermal treatment at 525, 575, and 625 °C is shown in Figure 2. Treatment of the glass at 525 °C reveals a dominant amorphous phase, while still containing nitrate impurities. The peaks at 2θ = ~43, 45, and 47 demonstrate the existence of nitrate phase attributable to JCPDS No. 00-038-944, 00-001-1215, and 00-345-1215, respectively^[^^25^^,^^26^^]^. Once the calcination temperature rises from 525 to 575 °C, the aforesaid nitrate phases undergo slow decomposition, and the resultant sample presents a high content of amorphous phase. New crystalline phases, including dicalcium silicate (JCPDS No. 00-024-0234) and calcium silicate (JCPDS 00-033-0303), appear and the glass-ceramic compound is produced with increasing the calcination temperature to 625 °C^[^^22^^,^^25^^]^.

**Fig. 3:**
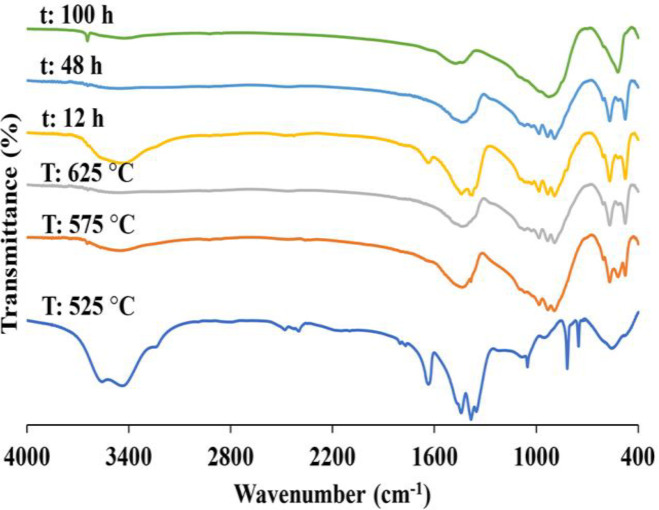
FTIR spectra of the glass powder obtained at different calcination temperature and gelation times

The FTIR spectra of the glass powder, when treating thermally at 525, 575, and 625 °C, are shown in Figure 3. Concerning the sample calcined at 525 °C, the bands observed at ~476 and ~568 belong to the symmetric bending of Si—O and symmetric stretching of P—O bond in the glass tetrahedron network, respectively. The peak at 894 cm^-1^ is attributable to the stretching vibration of Si—OH. The minor peaks situated at ~1049 and 1100 cm^-1 ^result from the P—O bond stretching vibrations, which are covered by the broad band in the range of 900-1250 cm^-1^, caused by stretching vibrations of Si—O—Si in silicate network. The wide peak at 1300-1600 cm^-1^ matches both N—O and C—O stretching vibrations in the remaining nitrate and adsorbed carbonate groups. The heat treatment from 525 to 625 °C led to the elimination of nitrate groups, reflecting the declined intensity of the aforesaid peak. The wide peak positioned at ~3450 cm^-1^ is ascribed to the stretching vibrations of O—H group in adsorbed water molecules and silanol (Si—OH) groups^[^^27^^,^^28^^]^. The FTIR spectra of the glass powders calcined at 575 and 625 °C display the unresolved bands, representative of amorphous phase. 

**Fig. 4:**
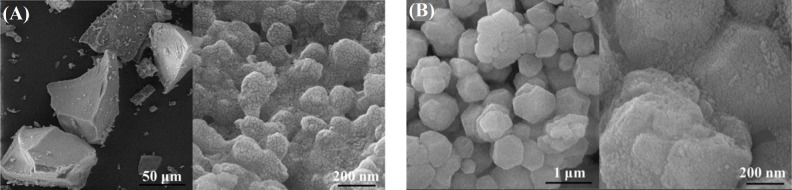
FESEM micrographs of the glass powder after heat treatment at (A) 575 and (B) 625 °C

The bands placed at ~476, 517, and 919 cm^-1 ^are related to the symmetric bending of Si—O, symmetric stretching of P—O bond, and stretching of cross-linked Si—O—Si in the glass tetrahedron network, respectively. The wide peak at ~3450 cm^-1^, along with a sharp one at 3644 cm^-1^, results from the stretching vibrations of O—H group in adsorbed water molecules and silanol (Si—OH) groups^[^^27^^-^^29^^]^.

Figure 4 depicts the FESEM micrographs of the glass powder at 575 and 625 °C treatments. The micrographs show the aggregates comprising of spherical nanoparticles in the range of 20-30 nm. Samples calcined at 575 °C generated deformed structures, displaying non-crystalline compounds with removed extra nitrates. Nanoscale structures are simply observable, covering the whole sample. The exit paths of denser water within the nanoscale structures are clearly observed in the Figure. Apparently, the glass powders, particularly those calcined at 625 °C, experience deformation to polygonal aggregates consisting of nanoscale structures, which suggests the development of a crystalline order.

The glasses produced by different gelation times (12, 48, and 100 h) were subjected to calcination at 575 °C and analysis by XRD, FTIR, and FESEM, to examine the impact of gelation time on nitrate phases. Theoretically, controlling the gelation time to avoid facile mass transfer within the colloid strands may create essential physical barriers against penetration. This control behavior will enable to govern the germination and, in particular, the development of nitrate structures or other materials of calcium phosphate phases in the course of synthesizing the glass. FTIR spectra of the glass powders at gelation times (12, 48, and 100 h), followed by calcination at 575 °C, depicted in Figure 3 reveals that once the gelation time increases from 12 to 100 h, two issues arise. First, the absorption bands belonging to the existence of calcium nitrate, namely 1300–1400 cm^-1^ and 1500–1600 cm^-1^ corresponding to bending and stretching vibrations of N—O bond, respectively, undergo gradual disappearance, which confirms the degradation of nitrate phase. Second, the peak at ~3560-3660 cm^-1 ^resulting from the stretching vibrations of O—H group silanol (Si—OH) groups slowly vanishes, which verifies the cross-linking of silanol groups, resulting in the creation of Si—O—Si network^[^^30^^,^^31^^]^. 

**Fig. 5:**
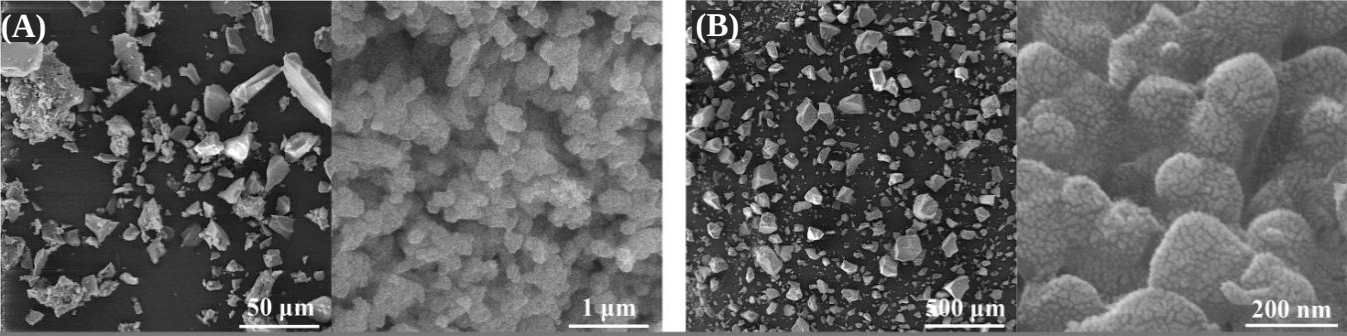
FESEM micrographs of glass powders obtained at gelation times of (A) 48 and (B) 100 h

Figure 5 exhibits the FESEM micrographs of glass powders fabricated at gelation times of 48 and 100 h, followed by calcination at 575 °C. Through comparison of the micrographs, it can be detected that: (i) all samples represent glass aggregates consisting of spherical nanoparticles, (ii) the nanometric structure has enlarged in the glass produced at a gelation time of 100 h compared to that of 48 h, and (iii) the glass constructed at a gelation time of 100 h yields further outflow paths of water molecules within nanoscale structure than the sample produced at a gelatin time of 48 h. This finding implies that the gelation time of 100 h has positive effects on the physical barricade to the germination and growth of amorphous glass phase. 

***In vitro*** **studies**

**Fig. 6:**
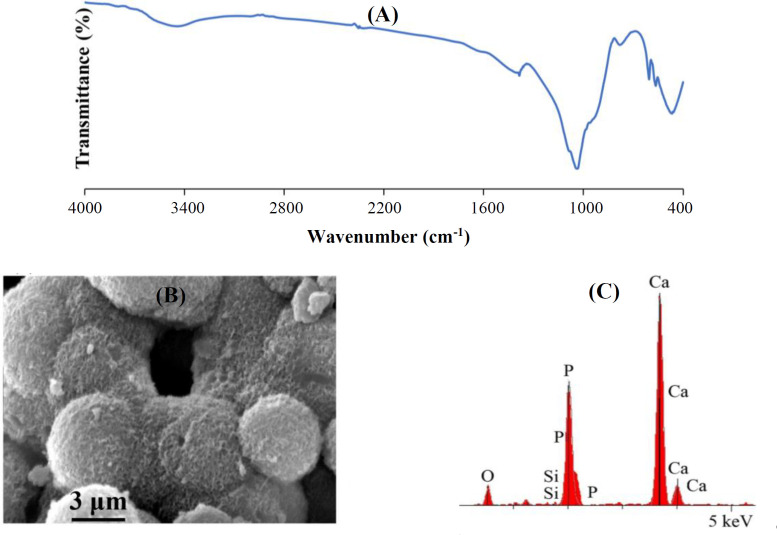
FTIR spectra (A), SEM micrograph (B), and EDS pattern (C) of bioactive glass powders after immersion in SBF for seven days. O, oxygen; P, phosphate; Si, silicon; Ca, calcium

The synthesis of bioactive glass yielded an optimized sample at the gelation time of 100 h and a calcination temperature of 575 °C, which was examined *in vitro*. Figure 6A depicts the FTIR spectra of glass powders after sopping in SBF for seven days. The representative apatite bands situated at 575 cm^-1^ and 604 cm^-1^ indexed to the asymmetric vibrations of P—O band are clear proofs for the creation of bioactive apatite layer on the surface of glass particles. In addition, an absorption band noticed at 1440 cm^-1^ belonging to the stretching vibrations of carbonate (${\mathrm{CO}}_{3}^{2-}$) groups, which displays the creation of carbonated apatite upon soaking in SBF. These observations suggest the proper bioactive activity of the manufactured glass powders^[^^30^^-^^33^^]^. Development of a shoulder at ~965 cm^-1^ attributable to P—O group proposes that the dissociation of non-bridge oxygen bonds, due to releasing Ca^2+^ ions into SBF after seven days, is associated with the creation of silanol groups in the intersection of glass with SBF, leading to the subsequent establishment of P—O bonds^[^^32^^]^. Figure 6B displays SEM micrographs prepared from the surface of bioactive glass powders after submersion in SBF for seven days. It is clear from the micrographs that a flake-shaped layer or cauliflower-like clusters is/are formed on the surface of the sintered powders after sopping in SBF for seven days, which mainly characterizes the bioactive apatite as established by FTIR analysis^[^^32^^,^^34^^]^. Figure 6C represents the EDS patterns recorded on the surface of glass powders after submersion in SBF for seven days. The patterns display the main peaks belonging to calcium and phosphorous with a calcium/phosphorous atomic ratio of 1.75, demonstrating that an apatite layer has been formed on the surface of the nanoparticles.

**Fig. 7:**
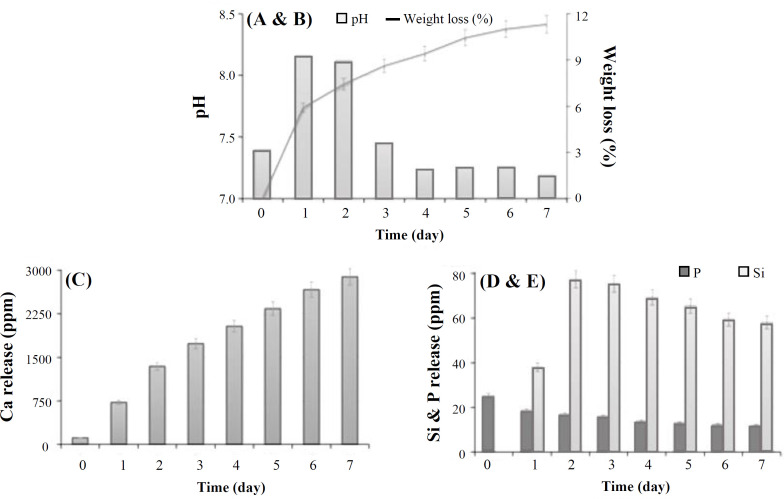
Weight loss (A) change in solution pH (B) and release trend of calcium (C), silicon (D), and phosphorous (E) during the immersion of glass powders in SBF

Figure 7A portrays the weight loss trend of glass powders after submersion in SBF for seven days, indicating that the sample degrades rapidly in the initial days of submersion in SBF, with gradual approach to a somewhat constant limit. The elevated weight loss at the early days of submersion results from the rapid ion exchange at the interface of glass and SBF solution and, subsequently, the formation of Si–OH group on the surface of glass particles. After a protective layer of apatite forms on the surface, the weight loss process halts reaching a rather constant value^[^^33^^,^^35^^]^, which is in accordance with pH variations (Fig. 7B). The Figure reveals a rise of pH parallel to the submersion time, due to releasing alkaline Ca^2+^ ions into the surrounding medium through exchanging with hydronium (H_3_O^+^) ions in the solution. Ion exchange decreases afterwards, and then pH stabilizes once bioactive apatite layers are formed on the surface of glass^[^^31^^]^. Figure 7C, 7D, and 7E displays the releasing trend of calcium, phosphorous, and silicon ions during the submersion of glass powder in SBF solution. As shown in the Figure, the concentrations of Ca^2+^ and silicon ions discharged into SBF solution rise continually with time at initial early days of sopping, followed by reaching a stable level. Two major parameters that control the concentrations of ions, particularly Ca^2+^ in the solution, are releasing ion from the bioactive glass and forming a bioactive apatite layer on the glass surface. When a bioactive apatite layer is absent in the initial days of sopping, the concentrations of ions undergo rises along with a rise in pH. The level of ion discharge into the surrounding medium drops once an apatite layer forms as a barricade against glass disintegration. A declining trend is visible regarding phosphorous, which is attributed to the phosphorus uptake by the glass surface during the apatite layer creation^[^^31^^,^^32^^]^. The release profile of Si^4+^ ion reveals a trend similar to Ca^2+^. However, the quantity of the released Ca^2+ ^is more than Si^4+ ^during the immersion time, owing to the high mobility of calcium ions within glass network. The cation exchange actually raises the concentration of hydroxyl in the solution, leading to the disintegration of the silica glass network. The release of soluble silica into the solution in the form of Si(OH)_4_ results from the breakdown of Si–O–Si bonds and the creation of Si–OH (silanols) groups at the glass-solution interface. The concentration of Si^4+ ^ion in the solution approaches a stable level when an apatite layer is formed on the glass surface with ceasing the disintegration of the glass^[^^31^^]^.

**Fig. 8:**
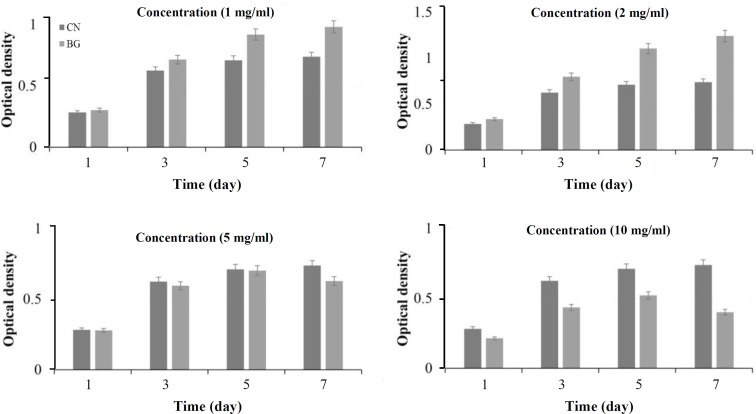
The optical density of hMSCs seeded on the bioactive glass (BG) powders with various concentrations. CN, control

Figure 8 represents the optical density of hMSCs subjected to different concentrations of bioactive glass powders (1.0, 2.0, 5.0, and 10.0 mg.mL^-1^) studied by MTT assay within seven days. According to the observations, when bioactive glass powders are present up to a concentration of 2.0 mg.mL^-1^, a significant increase in the power of differentiation occurs in hMSCs compared to the control sample. A rise in the glass concentration, on the other hand, led to the decreased viability of cells in comparison to the control. The ionic products obtained from the disintegration of bioactive glass could supply a sufficient stimulant for the cell growth and viability. 

Obviously, exceeding the ion concentration from a specific safe limit may result in cellular toxicity, as it is visible at glass concentrations of 5.0 and 10.0 mg.mL^-1[^^36^^,^^37^^]^.

**Fig. 9:**
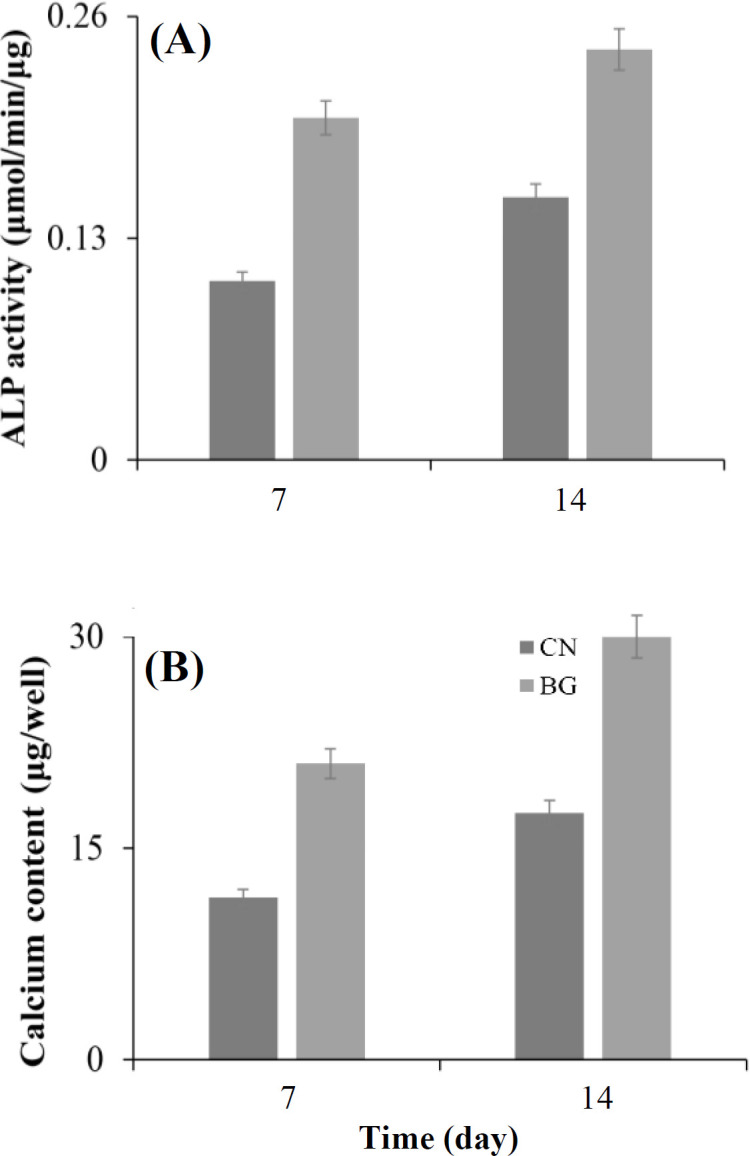
ALP activity (A) and quantified biomineralization ability of MSCs (B) in the presence of bioactive glass (BG) powders within 14 days. CN, control

A major factor in tissue engineering is the capacity of biomaterials to stimulate the differentiation of osteoblasts demonstrated by the elevated concentrations of ALP enzyme released by osteoblast cells^[^^38^^]^. Accordingly, ALP activity is known to be a marker for premature differentiation of osteoblast cells. The ALP activity of the bioactive glass powders within 14 days (Fig. 9A) reflected a significant difference (*p* < 0.05) between MSCs seeded with the control and without glass powders. As shown in the Figure, the ALP activity of MSCs represents continuous elevation when exposed to the bioactive glass powders, indicating the multiplication and differentiation of MSCs. Calcium biomineralization is known as a biomarker for the mature differentiation of osteoblasts and mineralization^[^^38^^]^. The results of calcium deposits measured within 14 days of osteogenic differentiation (Fig. 9B) demonstrate a significant increase in the biomineralization ability of MSCs (*p* < 0.05) once exposed to the bioactive glass powders. A rising trend of biomineralization is also observed in the Figure.

**Fig. 10:**
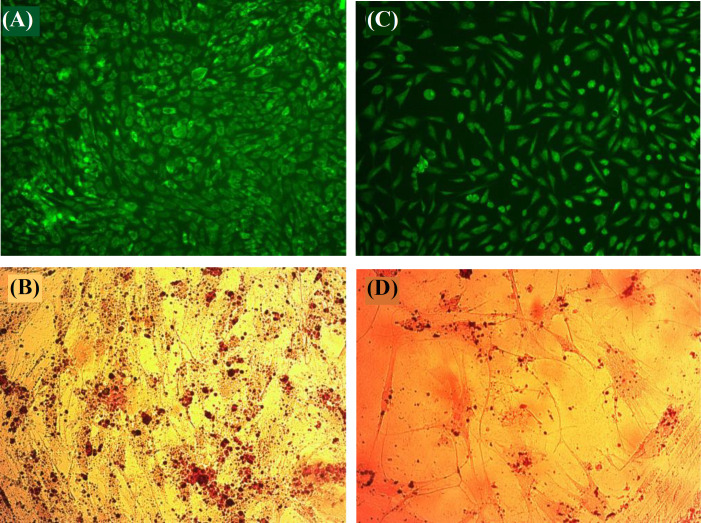
Light microscope micrographs of acridine orange (A) and Alizarin red-stained (B) hMSCs seeded on the bioactive glass powders, control without bioactive powders (C and D)

Figure 10A and 10C present the fluorescent images of hMSCs grown on the bioactive glass powders (with a concentration of 2.0 mg.mL^-1^) after staining by acridine orange during seven days. The viable cells are seen in green, and dead ones in red under the fluorescent light. According to the images, cell death did not occur, and the nucleus and cytoplasm of the cells were completely green. It is clear from the images that bioactive glass powders not only do not induce the cell death but also significantly improve the multiplication of cells (Fig. 10A) in comparison to the control (Fig. 10C). This behavior is attributable to the optimal ion discharge from glass powders, stimulating the cell multiplication and differentiation with no toxic consequences^[^^36^^]^. To investigate the hMSC differentiation, Alizarin red staining test was used as a qualitative tool for more confirmation of the biomineralization of MSCs. Reaction of calcium cation with alizarin dye occurred during the staining, forming chelation complexes, which can be observed as red spots under a light microscope^[^^38^^]^. The micrographs of the stained MSCs with (Fig. 10B) and without (Fig. 10D) bioactive glass powders reflect elevated volume of calcium deposits as a result of exposing the cells to glass powders, as verified by quantified biomineralization observations.

**Fig. 11:**
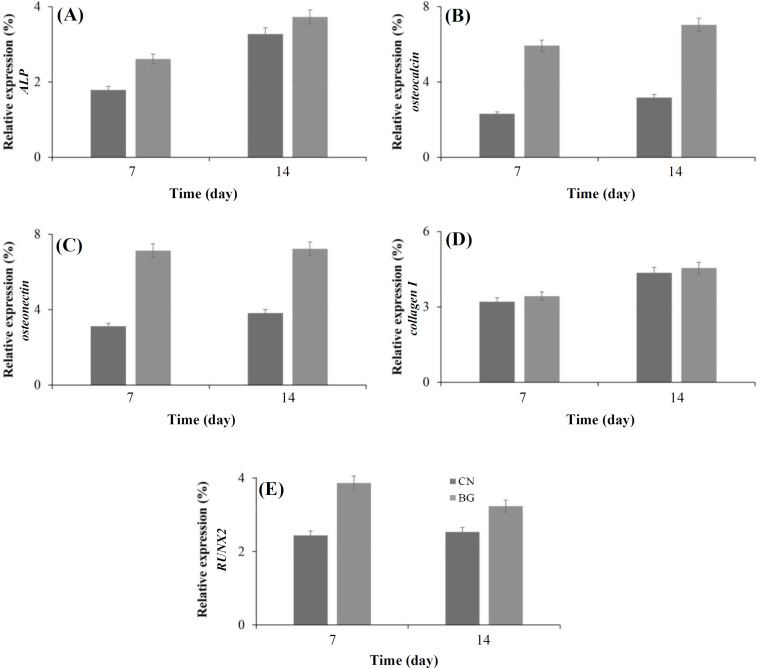
Relative gene expression levels of *ALP* (A), *osteocalcin* (B), *osteonectin* (C), *collagen I* (D), and *RUNX2* (e) within 14 days. CN, control

MSCs undergo differentiation into osteoblast lineage during the osteogenesis process via multiple maturation phases in arranged time arrays and ultimately into an osteoblast phenotype with full activation. A number of transcription factors and osteogenic-related genes contribute to the osteogenesis, including *ALP*, *collagen I*, *osteocalcin*, *osteonectin*, and *RUNX2*^[^^38^^]^. Figure 11 depicts the gene expression pattern of five different genes, namely *ALP*, *osteocalcin*, *osteonectin*, *collagen I*, and *RUNX2* within 14 days of cell differentiation. Qualitative analysis of osteoblast gene expression indicated the stimulatory effect of glass particles in osteogenic differentiation and expression of the target genes. Osteogenesis occurs in three stages of osteoblast differentiation, matrix formation, and matrix mineralization.

ALP is the marker of the mature osteoblastic phenotype and extracellular matrix maturation. *RUNX2* acts as a master gene in osteogenic differentiation and regulates the expression of downstream transcription factors and osteogenic markers. As stated by Komori *et al.*^[^^39^^]^, *ALP* gene expression occurs at the early and middle of the above stages and *RUNX2* expression varies during the three steps. They found that *RUNX2* is a main regulator of osteoblast differentiation and has a high expression level during the early differentiation stage, but it is not sufficient alone^[^^40^^]^. According to our results, *ALP* gene expression in glass-treated sample increased markedly after 7 and 14 days of culture compared with the control sample. *RUNX2* had a rising trend at seven days post culture, i.e. at the early stages of the gene expression, but the uptrend decreased afterward until the 14^th^ day. 

Sundaramurthi *et al.*^[^^41^^]^ have reported that the *osteocalcin* and *osteonectin* genes play important roles in the mineralization and early formation of calcium crystals. *Osteocalcin* is a non-collagen protein expressed by mature osteoblasts. This compound consists of three carboxyglutamic acid residues^[^^39^^-^^42^^]^. *Osteonectin* is a glycoprotein that initiates the mineralization and promotes crystal formation. It has a high binding affinity for tissue calcium and collagen^[^^42^^]^. The *osteocalcin* and *osteonectin* expression levels of the bioactive glass were significantly different (more than twofold) from the control at 7 and 14 days of differentiation; *osteonectin* expression level was almost unaltered throughout the test period. 

Collagen I is the dominant collagen type in bone and the framework of structural protein of matrices for inorganic deposition. The Collagen I encoding gene also showed more expression at 7 and 14 days of osteoblast ifferentiation compared with the control sample. The data reveal that the treatment of MSCs with bioactive glass powders leads to significant rises in the expression of all genes. The particle size, composition, surface chemistry, dissolution rate, and released ions are main parameters influencing the cell growth, differentiation, and gene expression. The bioactive glass powders (SiO_2_ and P_2_O_5_) act as network-making factors for cell hosting and adhesion. Moreover, silanol groups (Si—OH), created by the exchange of Ca^2+^ with H_3_O^+^, can positively affect the growth and adhesion of MSCs. Besides, Si^4+^ and Ca^2+^ discharged from the bioactive glass into the cell culture medium have been reported to have a significant contribution to cell multiplication and differentiation, which in turn stimulate the high levels of gene expression^[^^43^^,^^44^^]^.

Synthesis of bioactive glass powders in the ternary system of SiO_2_−CaO−P_2_O_5_ (58:38:4%mol.) was performed by sol–gel technique. The impacts of gelation and calcination temperature on the microstructure and chemical composition of the glasses were examined using XRD, FESEM, EDS, and FTIR. According to our findings, the gelation time has an effect on the physical barricade to germination and growth of amorphous glass phase. Additionally, nitrate impurities can be completely removed upon rising the calcination temperature. *In vitro* tests in SBF were conducted on as-synthesized bioactive glasses. The observations of SEM micrographs, MTT cytotoxicity, cell staining, ALP activity, biomineralization, and gene expression tests demonstrated favorable bioactivity, biocompatibility, and osteogenic capacities of the synthesized bioactive glass powders. 

Overall, this study showed that a gelation time of 100 h and a calcination temperature of 575 °C are optimal conditions for the synthesis of highly pure nitrate-free bioactive glass nanopowders with improved bioactivity and osteogenic properties as demanded for biomedical engineering.
